# Uptake of methodological advances for synthesis of continuous and time-to-event outcomes would maximize use of the evidence base

**DOI:** 10.1016/j.jclinepi.2020.05.010

**Published:** 2020-08

**Authors:** Suzanne C. Freeman, Alex J. Sutton, Nicola J. Cooper

**Affiliations:** Department of Health Sciences, University of Leicester, Leicester LE1 7RH, UK

**Keywords:** Continuous outcomes, Health technology assessment, Time-to-event outcomes, Evidence synthesis, Decision models, Clinical decision-making

## Abstract

**Objective:**

The objective of the study is to establish how often continuous and time-to-event outcomes are synthesized in health technology assessment (HTA), the statistical methods and software used in their analysis and how often evidence synthesis informs decision models.

**Study Design and Setting:**

This is a review of National Institute of Health Research HTA reports, National Institute for Health and Care Excellence (NICE) technology appraisals, and NICE guidelines reporting quantitative meta-analysis or network meta-analysis of at least one continuous or time-to-event outcome published from April 01, 2018 to March 31, 2019.

**Results:**

We identified 47 eligible articles. At least one continuous or time-to-event outcome was synthesized in 51% and 55% of articles, respectively. Evidence synthesis results informed decision models in two-thirds of articles. The review and expert knowledge identified five areas where methodology is available for improving the synthesis of continuous and time-to-event outcomes: i) outcomes reported on multiple scales, ii) reporting of multiple related outcomes, iii) appropriateness of the additive scale, iv) reporting of multiple time points, and v) nonproportional hazards. We identified three anticipated barriers to the uptake and implementation of these methods: i) statistical expertise, ii) software, and iii) reporting of trials.

**Conclusion:**

Continuous and time-to-event outcomes are routinely reported in HTA. However, increased uptake of methodological advances could maximize the evidence base used to inform the decision making process.

**What is new?**

**Key findings**
• Through a review of NIHR HTA reports, NICE technology appraisals, and NICE guidelines published between 1st April 2018 and 31st March 2019, we establish that continuous and time-to-event outcomes are routinely synthesized within HTA articles. However, only two-thirds of articles used the results from an evidence synthesis of continuous or time-to-event outcomes to inform an economic decision model.
• Evidence from the review combined with expert knowledge of the methodological field identified five key areas where underused methodology is available for improving the synthesis of continuous and time-to-event outcomes: i) outcomes reported on multiple scales, ii) reporting of multiple related outcomes, iii) appropriateness of the additive scale, iv) reporting of multiple time points, and v) nonproportional hazards.

**What this adds to what was known?**
• We identified three anticipated barriers to the uptake and implementation of these methods: i) availability of specialist statistical expertise for model selection, model fitting, and interpretation of results, ii) user-friendly software for implementing complex statistical and/or nonstandard models, and iii) limited reporting of individual trials.

**What is the implication and what should change now?**
• More research is needed to develop, refine, and generalize where possible methods for synthesizing continuous and time-to-event outcomes for the purpose of decision modeling to maximize the evidence base used in the decision making process.

## 1 Introduction

Health technology assessment (HTA) is a form of policy research that examines the short- and long-term consequences related to the use of a health technology. The purpose of HTA is to facilitate decision making by providing information about the most clinically effective and cost-effective interventions for a given condition and to provide this information in a systematic, transparent, and unbiased manner. Evaluating health technologies using this evidence-based approach is a key component of health care decision making often resulting in cost savings and better quality of treatment for patients [1].

Evidence synthesis is a well-established component of HTA applied to quantitatively combine the data from multiple trials to obtain an overall pooled estimate(s) of clinical effectiveness, which may be used to inform an associated economic evaluation. For comparisons between two health care interventions, it is common practice to apply pairwise meta-analysis (MA) methods to obtain pooled effectiveness estimates; however, where more than two interventions are of interest, network meta-analysis (NMA) [2] (also known as multiple treatment comparisons [3] or mixed treatment comparisons [4]) is applied. NMA extends pairwise MA to allow the simultaneous estimation of comparative effectiveness of multiple interventions using an evidence base of trials that individually may not compare all intervention options, but together form a connected network of comparisons.

Since 2004, in the United Kingdom, the National Institute for Health and Care Excellence (NICE) has recommended MA of randomized controlled trials (RCTs) as its preferred method for evidence synthesis [5]. In 2013, this recommendation was updated to acknowledge the role of NMA methods for assessing clinical effectiveness from all relevant studies reporting clinically relevant outcomes [6]. It is a combination of the evidence on clinical effectiveness from evidence synthesis along with economic decision models which form the basis of NICE guidance for improving health and social care in the United Kingdom [1].

The synthesis of continuous and time-to-event outcomes is often perceived to be more complex than the synthesis of binary outcomes because of heterogeneous reporting of outcomes across trials, a higher propensity for outcomes to be missing, and difficult interpretability of results. In this article, we start by conducting a review of National Institute of Health Research (NIHR) HTA reports, NICE technology appraisals, and NICE guidelines to establish the current state of play with regard to how often continuous and time-to-event outcomes are reported in HTA, the statistical methods and software used in practice for the synthesis of continuous and time-to-event outcomes, and how much of the evidence from evidence syntheses of continuous and time-to-event outcomes contributes to the economic decision model. From the review and expert methodological knowledge, we identify key challenges in synthesizing continuous and time-to-event outcomes and provide examples of methodological advances to overcome these issues as well as identifying potential barriers to the implementation and uptake of these methods.

## 2 Review of NICE technology appraisals, NICE guidelines, and NIHR HTA reports

### 2.1 Methods

We conducted a review of NICE technology appraisals, NICE guidelines, and NIHR HTA reports published between April 1, 2018 and March 31, 2019. Articles were considered eligible for inclusion in this review if they contained a quantitative MA or NMA for at least one continuous or time-to-event outcome. Articles were excluded if they reported diagnostic outcomes, prognostic outcomes, and feasibility studies or were updates of previous reviews without evidence synthesis. NICE technology appraisals and NICE guidelines were identified from the lists of published technology appraisals and guidelines on the NICE website (https://www.nice.org.uk/guidance/published?type=ta and https://www.nice.org.uk/guidance/published?type=apg,csg,cg,mpg,ph,sg,sc). NIHR HTA reports were identified from the NIHR HTA website (https://www.journalslibrary.nihr.ac.uk/hta/volume/?volume=22#/ and https://www.journalslibrary.nihr.ac.uk/hta/volume/?volume=23#/). Articles were screened for eligibility by one author (S.C.F.). Articles where eligibility was unclear were discussed by all authors.

We extracted details relating to the outcomes and analysis methods for evidence synthesis of clinical data, details relating to the use of a decision model, and how this may have been informed by an evidence synthesis and software. Informed by our expert methodological knowledge, we specifically extracted details relating to analysis of multiple outcomes, multiple time points, and standardization to determine whether recent advances in evidence synthesis methodology, to address these common issues, have filtered through to the HTA process. Items that were prespecified for extraction are listed in Appendix A. Based on our knowledge of the new methodologies, we identified what we anticipate to be three barriers to implementing these methods.

For NICE guidelines, which often consist of multiple evidence reviews, we included the first evidence review, which reported both an eligible evidence synthesis and an economic analysis. If none of the evidence reviews included an economic analysis, then the first evidence review to consider an eligible evidence synthesis was included. Any guideline that did not include a methods section for evidence synthesis was excluded from this review.

For NICE technology appraisals, which often consist of multiple documents from committee meetings, we only extracted data from the company submission document. Where this indicated the presence of additional appendices containing further details about the evidence synthesis methodology, we requested the appendices from NICE. A total of 25 appendices were requested with 10 appendices received. Terminated appraisals were excluded from this review.

### 2.2 Results

**Fig. 1:**
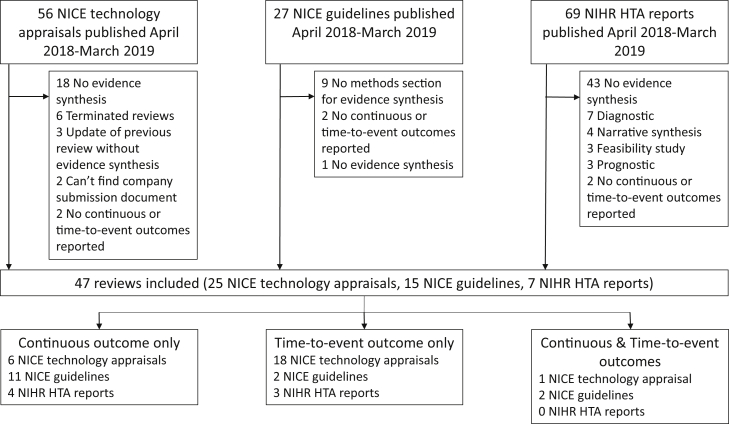
Flowchart of article selection process. HTA, health technology assessment; NICE, National Institute for Health and Care Excellence; NIHR, National Institute for Health Research.

Between April 1, 2018 and March 31, 2019, 56 NICE technology appraisals, 27 NICE guidelines, and 69 NIHR HTA reports were published. A total of 47 articles were considered eligible for this review of which 25 (of 56, 45%) were NICE technology appraisals, 15 (of 27, 56%) were NICE guidelines, and 7 (of 69, 10%) were NIHR HTA reports (Fig. 1). A list of the articles included in this review can be found in Appendix B.

Continuous and time-to-event outcomes were routinely reported with 51% (24) of articles reviewed reporting at least one continuous outcome and 55% (26) of articles reporting at least one time-to-event outcome. Three articles reported both continuous and time-to-event outcomes. The results for continuous outcomes are presented in Table 1 and for time-to-event outcomes in Table 2 with key points discussed below.

**Table 1:** Results for continuous outcomes

Characteristic	NICE technology appraisal	NICE guidelines	NIHR HTA report
No. of articles	7	13	4
Clinical field			
Cardiovascular	0	1 (8%)	1 (25%)
Dementia	0	1 (8%)	1 (25%)
Kidney	0	2 (15%)	0
Mental health	0	2 (15%)	0
Psoriatic arthritis	2 (29%)	0	0
Respiratory	1 (14%)	1 (8%)	0
Skin	2 (29%)	0	0
Cerebral palsy	0	1 (8%)	0
Diabetes	1 (14%)	0	0
Hearing loss	0	1 (8%)	0
Lyme disease	0	1 (8%)	0
Obesity	0	0	1 (25%)
Oncology	1 (14%)	0	0
Pancreatitis	0	1 (8%)	0
Pharmacy	0	1 (8%)	0
Rheumatoid arthritis	0	1 (8%)	0
Sleep	0	0	1 (25%)
Type of intervention			
Pharmacological	7 (100%)	4 (31%)	0
Nonpharmacological	0	4 (31%)	2 (50%)
Both	0	5 (38%)	2 (50%)
Synthesis			
Method of synthesis			
Meta-analysis	1 (14%)	13 (100%)	4 (100%)
Network meta-analysis	7 (100%)	3 (23%)	0
Outcome measure			
Mean difference	1 (14%)	11 (85%)	2 (50%)
Standardized mean difference	1 (14%)	1 (8%)	1 (25%)
Median difference	1 (14%)	0	0
Mean	1 (14%)	0	0
Percentage	2 (29%)	0	1 (25%)
Risk difference	1 (14%)	0	0
Relative risk	0	1 (8%)	0
Fixed effect or random effects			
Fixed effect	0	1 (8%)	0
Random effects	0	1 (8%)	4 (100%)
Both	4 (57%)	11 (85%)	0
Unclear	3 (43%)	0	0
Analysis details			
Multiple outcomes simultaneously	0	0	0
Lumping of interventions	0	0	1 (25%)
Multiple pairwise comparisons	1 (14%)	9 (69%)	1 (25%)
Standardization of any outcomes	1 (14%)	5 (38%)	2 (50%)
Multiple time points	1 (14%)	7 (54%)	1 (25%)
Presentation of results			
Forest plot	1 (14%)	13 (100%)	3 (75%)
Network diagram (if NMA only)	4/7 (57%)	2/3 (67%)	0/0
Tables	6 (86%)	2 (15%)	3 (75%)
Other	1 (14%)	0	0
Software for synthesis			
Not reported	4 (57%)	0	1 (25%)
R & WinBUGS	1 (14%)	0	0
Cochrane Review Manager	0	10 (77%)	2 (50%)
Cochrane Review Manager & R	0	1 (8%)	0
Cochrane Review Manager & WinBUGS	0	2 (15%)	0
Stata	1 (14%)	0	1 (25%)
Stata & WinBUGS	1 (14%)	0	0
Decision model			
Decision model details			
Decision model	6 (86%)	8 (62%)	3 (75%)
Graph of model structure	5/6 (83%)	7/8 (88%)	1/3 (33%)
Clinical results inform the decision model	6/6 (100%)	7/8 (88%)	3/3 (100%)
How much clinical evidence informs the decision model?			
Meta-analysis	0	2/8 (25%)	2/3 (67%)
Network meta-analysis	5/6 (83%)	2/8 (25%)	0
Not reported	0	1/8 (13%)	0
Single trial	1/6 (17%)	3/8 (38%)	1/3 (33%)
Analysis method in the decision model			
Cost comparison	1/6 (17%)	0	0
Decision tree followed by Markov model	1/6 (17%)	2/8 (25%)	0
Discrete event simulation	0	0	1/3 (33%)
Markov model	2/6 (33%)	3/8 (38%)	0
Multistate model	1/6 (17%)	2/8 (25%)	0
Partitioned survival	1/6 (17%)	0	0
UK Health Forum microsimulation	0	0	1/3 (33%)
Cost–utility analysis (model unspecified)	0/6	1/8 (13%)	1/3 (33%)
Software for cost-effectiveness			
Excel	2 (33%)	3 (38%)	1 (33%)
Not reported	3 (50%)	5 (63%)	0
R	0	0	1 (33%)
SAS	0	0	1 (33%)
Visual Basic & Excel	1 (17%)	0	0

**Table 2:** Results for time-to-event outcomes

Characteristic	NICE technology appraisal	NICE guidelines	NIHR HTA report
No. of articles	19	4	3
Clinical field			
Oncology	18 (95%)	2 (50%)	3 (100%)
Cardiovascular	0	1 (25%)	0
Kidney	0	1 (25%)	0
Multiple sclerosis	1 (5%)	0	0
Type of intervention			
Pharmacological	19 (100%)	2 (50%)	2 (67%)
Nonpharmacological	0	1 (25%)	1 (33%)
Both	0	1 (25%)	0
Synthesis			
Method of synthesis			
Meta-analysis	4 (21%)	4 (100%)	1 (33%)
Network meta-analysis	16 (84%)	0	3 (100%)
Outcome measure			
Fractional polynomial	1 (5%)	0	0
Hazard ratio & fractional polynomial	1 (5%)	0	0
Hazard ratio	11 (58%)	4 (100%)	2 (67%)
Hazard ratio (PH & time-varying)	1 (5%)	0	0
Mean survival	1 (5%)	0	0
Median survival	2 (11%)	0	0
Median survival & hazard ratio	1 (5%)	0	0
Relative risk	0	0	1 (33%)
Time varying hazard ratio	1 (5%)	0	0
Fixed effect or random effects			
Fixed effect	2 (11%)	0	0
Random effects	0	0	1 (33%)
Both	8 (42%)	4 (100%)	1 (33%)
Unclear	9 (47%)	0	1 (33%)
Analysis details			
Multiple outcomes simultaneously	0	0	0
Lumping of interventions	1 (5%)	0	0
Multiple pairwise comparisons	3 (16%)	4 (100%)	1 (33%)
Multiple time points	1 (5%)	2 (50%)	0
Standardization of any outcomes	1 (5%)	1 (25%)	0
Presentation of results			
Forest plot	7 (37%)	4 (100%)	2 (67%)
Network diagram (if NMA only)	11/16 (69%)	0/0	3/3 (100%)
Tables	13 (68%)	0	3 (100%)
Kaplan–Meier	10 (53%)	0	0
Other	3 (16%)	0	0
Software for synthesis			
Not reported	14 (74%)	0	1 (33%)
OpenBUGS	1 (5%)	0	0
R	1 (5%)	0	0
R & WinBUGS	1 (5%)	0	0
Cochrane Review Manager	0	4 (100%)	0
Stata	1 (5%)	0	0
Stata, R & JAGS	0	0	1 (33%)
WinBUGS	1 (5%)	0	1 (33%)
Decision model			
Decision model details			
Decision model included	19 (100%)	4 (100%)	3 (100%)
Graph of model structure	19 (100%)	4 (100%)	3 (100%)
Clinical results inform the decision model	19 (100%)	4 (100%)	3 (100%)
How much clinical evidence informs the decision model?			
Historical control	1 (5%)	0	0
Meta-analysis	2 (11%)	2 (50%)	1 (33%)
Network meta-analysis	12 (63%)	0	0
Single trial	4 (21%)	2 (50%)	2 (67%)
Analysis method in the decision model			
Decision tree followed by Markov model	2 (11%)	1 (25%)	0
Markov model	1 (5%)	1 (25%)	0
Multi-state model	1 (5%)	0	0
Partitioned survival	14 (74%)	2 (50%)	2 (67%)
Semi-Markov cohort	1 (5%)	0	0
Weibull	0	0	1 (33%)
Software for cost-effectiveness			
Excel	9 (47%)	2 (50%)	3 (100%)
Not reported	10 (53%)	2 (50%)	0

NMA, network meta-analysis; PH, proportional hazards.

#### 2.2.1 Continuous outcomes: which methods of evidence synthesis are used in practice?

Pairwise MA was reported for all NICE guidelines and NIHR HTA reports, whereas NMA was reported in all NICE technology appraisals. Across all reviews, the most common outcome measure (for the first reported continuous outcome) was the mean difference (MD). Alternative outcome measures included standardized mean difference (SMD), mean, percentage, risk difference, and relative risk. Articles reporting continuous outcomes covered a wide range of clinical fields and included both pharmacological and nonpharmacological interventions. As such, a wide range of outcomes were considered including disease-specific scales (e.g., Alzheimer's disease assessment scale) and truly continuous measures (e.g., weight change, Appendix Table C1).

Nine (69%) NICE guidelines reported multiple pairwise comparisons and one NIHR HTA report of pairwise MA lumped interventions together. Statistical methods to analyze multiple outcomes simultaneously were not reported in any articles. Multiple time points were reported in nine articles and were handled by reporting separate meta-analyses at specific time points. In total, only eight reviews (33%) used standardization of any reported continuous outcomes.

All NICE guidelines and two NIHR HTA reports used Cochrane Review Manager (RevMan) to synthesize clinical evidence. Alternative software options included R, Stata, and WinBUGS. Where reported, the most commonly used software for cost effectiveness was Microsoft Excel.

#### 2.2.2 Time-to-event outcomes: which methods of evidence synthesis are used in practice?

All NICE guidelines reported pairwise MA, whereas NMA was more frequently reported in NICE technology appraisals and NIHR HTA reports. Most articles were pharmacological interventions in oncology. The most common outcomes were overall and progression-free survival (Appendix Table C2). As such, the most common outcome measure for reporting time-to-event outcomes was the hazard ratio. Alternative outcome measures included time-varying hazard ratios, fractional polynomial coefficients, median survival, mean survival, and relative risk.

Multiple pairwise comparisons were reported in eight articles (31%), and in one NICE technology appraisal, two drug interventions were lumped together for inclusion in the NMA alongside eight other interventions. Combining these two interventions into one treatment node for the NMA assumes that both interventions have the same efficacy across trials for the endpoints assessed. Multiple time points were reported in three articles and in all articles were handled by reporting separate meta-analyses at specific time points. No articles reported analyzing multiple outcomes simultaneously.

Reporting of software used for evidence synthesis of clinical data was poor for NICE technology appraisals. As with continuous outcomes, all NICE guidelines reported using Cochrane Review Manager (RevMan) to synthesize the clinical evidence and alternative software options included R, Stata, and WinBUGS. Where reported, Microsoft Excel was used for cost-effectiveness analyses.

#### 2.2.3 Continuous outcomes: how do the evidence synthesis results inform the economic decision models?

Decision models were reported in the majority of articles across all review types for continuous outcomes (17, 71%). The decision models were informed by clinical results in 16 reviews, and of these, 11 (69%) were informed by the evidence synthesis result. The decision models in the remaining five trials were informed by single trials only.

#### 2.2.4 Time-to-event outcomes: how do the evidence synthesis results inform the economic decision models?

All articles reporting a time-to-event outcome reported a decision model, with all articles reporting that clinical results were used to inform the decision model. Overall, the decision models in most articles were informed by evidence synthesis results (17, 65%). However, half of the NICE guidelines, four NICE technology appraisals, and two NIHR HTA reports were informed by a single trial despite an evidence synthesis being conducted. Reasons for only using a single trial ranged from article-specific considerations such as “only one study measured health-related quality of life in UK patients with nonsmall-cell lung cancer” to unclear statements such as “one study identified as most suitable”.

## 3 Challenges in synthesizing continuous and time-to-event outcomes

In the previously mentioned review, MA was often conducted separately for each time point and/or outcome. This approach to analysis lacks coherence as a different evidence base is used in each analysis and restricts the usefulness of the evidence synthesis for informing the decision model. It would be better to extend the evidence synthesis methodology to allow for multiple outcomes and/or multiple time points (as well as multiple treatments). In this way, the evidence base used for decision making can be maximized. Below we highlight five of the key challenges for synthesizing continuous and time-to-event outcomes and describe some of the methods available to overcome these challenges and maximize the evidence base available. We highlight key examples from the review itself and draw on the literature to promote and illustrate their potential for improving syntheses of continuous and time-to-event outcomes in the future.

### 3.1 Outcomes reported on multiple scales

For continuous outcomes, trials may individually measure an outcome on multiple instruments, whereas only one is chosen to contribute to the synthesis. This can mean that some outcome data are excluded from the MA [7]. To avoid excluding data in clinical areas, such as pain and anxiety, where outcomes are frequently measured across multiple scales, they are often analyzed as SMD. However, SMD is hard to interpret in a meaningful way and is limited in its ability to be included in decision models [8]. Methods to overcome the problems with SMD include transformation back to one of the original scales or conversion to relative risk [9].

In our review of NIHR HTA reports, NICE technology appraisals, and NICE guidelines, we identified one NICE guideline which reported an NMA of SMD and included the result of the NMA in the decision model [10]. NG116 reported an NMA of changes in posttraumatic stress disorder (PTSD) symptoms between baseline and treatment endpoint with alternative scenarios considering change in PTSD symptoms between baseline and 1–4 month follow-up. The decision model required probabilities of remission for each treatment which could not be directly estimated from the SMD. Therefore, SMD was transformed into a log odds ratio and then exponentiated into an odds ratio from which the probability of remission for each intervention was calculated. Converting SMD to a log odds ratio requires the assumption that there is an arbitrary cutoff point on the underlying scale for determining whether a treatment is effective or not. The disadvantages of dichotomizing continuous outcomes in this way have been well documented [[11], [12], [13]]. However, by making this assumption, the authors were able to make use of all the available evidence.

When data are skewed, methods for combining medians have been shown to perform better than SMD [14]. However, another alternative to standardization is mapping to a common scale. This is a method of synthesis on ratios of different outcome scales [7,15,16] which allows data on all scales to be synthesized simultaneously and contribute to a single outcome expressed on each of the native scales. An advantage of this approach in a decision making scenario is that an appropriate scale for inclusion in the economic model can be easily chosen. Lu et al. [16] fit these models in a Bayesian framework using WinBUGS. WinBUGS is a specialist statistical software package requiring specialist statistical expertise to ensure appropriate prior distributions are selected and the models parameters and results are interpreted correctly. Both the choice of software and the need for specialist statistical expertise are likely to be barriers to the uptake of this method. In our review of NIHR HTA reports, NICE technology appraisals, and NICE guidelines, all NICE guidelines reported pairwise MA using RevMan to conduct their analyses. RevMan is limited in its analysis capabilities and uses a frequentist approach to analysis. To encourage the uptake of these methods within HTA articles, it may be important to provide software training alongside training on the methods themselves.

### 3.2 Reporting of multiple related outcomes

For both continuous and time-to-event outcomes, multiple separate meta-analyses of related outcomes are undesirable because they limit the evidence base used and often hinder the exploration of important issues in the clinical area such as how intervention effects change over time and how different outcome scales relate to one another. This is particularly pertinent (although not exclusive) to the NMA setting where different interventions may have been evaluated using different outcome measures and thus choosing specific outcomes causes systematic exclusion.

Multivariate MA can allow the synthesis of multiple outcomes on their original scales and account for multiple correlated outcomes, which are often reported in RCTs, for example, systolic and diastolic blood pressure or progression-free survival and overall survival [17]. This avoids the need to exclude any outcome data and means that multiple outcomes can be synthesized on their original scales. Accounting for the correlation between outcomes can result in increased precision in treatment effect estimates [18,19]. However, multivariate MA models become increasingly difficult to fit as data on each scale become sparse and/or the number of outcome scales used increases [17,20,21]. Multivariate MA models can be extended to the NMA setting [22]. Barriers to the uptake of multivariate MA and NMA are likely to include the need for specialist statistical expertise for both the fitting of the model and the interpretation of the model results as well as the need for software beyond RevMan. A recently published NICE technical support document on multivariate MA may help make this methodology more accessible to the HTA audience [23].

An NMA of antivirals for the treatment of influenza is an example of an NMA specifically developed for NICE to identify which antiviral was most effective at preventing influenza but for which the relevant trials reported different outcomes [24]. The network consisted of three antivirals each directly compared with a placebo but without any trials directly comparing antivirals to each other. In the analysis of individual studies, studies reported either time to alleviation of fever, time to alleviation of symptoms, or both outcomes. Synthesis of these studies was further inhibited by the reporting of different summary measures, for example, mean time, median time, and proportion symptom free at the end of follow-up. To overcome these complexities, an NMA model consisting of a Weibull model with exchangeable treatment effects that were independent for each outcome but had a common random effect mean for the two outcomes was fitted [24]. The advantage of this approach was the simultaneous synthesis of two outcomes estimating a single summary measure allowing conclusions to be drawn about the comparative efficacy of four treatments for which head-to-head trials did not exist and preventing the need to either exclude relevant trials or conduct multiple MA. In a decision making scenario, the advantage of this approach is that the single Weibull model parameter estimated can be easily incorporated within a decision model.

### 3.3 Appropriateness of the additive scale

Previous research for the synthesis of binary outcomes has focused on the appropriateness of different outcome scales (e.g., odds ratios vs. relative risks vs. risk differences) [25]. However, for continuous outcomes, additive scales have, almost unquestionably, been used (i.e., differences in means between groups). In recent MA of continuous outcomes, particularly in the areas of pain and depression [[26], [27], [28], [29]], between-study heterogeneity has been explained (in part) by the inclusion of covariates to account for differences between trials in the severity of pain/depression at baseline. In this case, an additive scale and hence the use of MD and SMD may not be appropriate. An alternative outcome measure on a multiplicative scale, the ratio of means, has been proposed in a series of recent articles [[30], [31], [32]]. Using a multiplicative scale avoids the strong assumption that all tests are linear transformations of the same underlying measurement scale. In addition, the ratio of means does not require knowledge of the pooled standard deviation, which is required for SMD but is often unknown by clinicians, and therefore has a more natural clinical interpretation [30,31]. Furthermore, in contrast to the SMD, the ratio of means provides a direct probability that can easily be incorporated within a decision model. However, in our review of NIHR HTA reports, NICE technology appraisals, and NICE guidelines, we found no articles reporting the ratio of means. Despite being a fairly straightforward method to implement, requiring only the mean outcome from each treatment arm, there appears to be a lack of knowledge about the ratio of means, the assumptions it makes, and when it may be appropriate to use. Uptake of the ratio of means may increase over the next few years after its addition to the Cochrane Handbook for Systematic Reviews of Interventions (Chapter 6.5.1.3) where it is recommended as an effect measure for outcomes which are physical measurements taking only positive values [33].

### 3.4 Multiple time points

For both continuous and time-to-event outcomes, appropriately dealing with multiple time points is especially important in the HTA setting, as a key component of decision models is modeling effectiveness over the full follow-up duration, so it is essential that any trends over time are incorporated within the decision model. RCTs often report the same outcomes at multiple time points, for example, pain at 2, 4, and 8 weeks postoperation. However, studies that do not report the outcome of interest at the specific time point of interest can be excluded from the synthesis. As a result, several methods to account for multiple time points have been proposed in the literature [[34], [35], [36]] with a recent review comparing three of these methods [37]. Methods for accounting for multiple time points depend heavily on the time points reported in the individual trials and are likely to require specialist statistical expertise for the interpretation of results and software beyond RevMan.

Lu et al. [38] accounted for outcomes reported at multiple time points using a piecewise exponential model. The network consisted of six treatments and 41 RCTs with RCTs reporting the outcome of interest and healing rate, at least once at 4, 6, 8, or 12 weeks. At each time point, the network diagram took on a different shape reflecting the variable evidence base available. An NMA model was fitted in which the time horizon was divided into four time periods with a piecewise hazard for each time period. A treatment effect was placed on time period 1 with treatment effects in subsequent time periods related to period 1 via a random walk model, which assumed that treatment effects were similar in each time period. The between-trial correlations between the treatment effects at different time points are implicit in the random walk variance. This is an example of simultaneously ‘borrowing strength’ on comparative effects across time periods while combining direct and indirect evidence on treatment comparisons within each time period. The model produced a hazard ratio for each treatment effect for each time period. This example demonstrates the potential for NMA methodology to account for multiple time points allowing the synthesis of a greater proportion of data from the evidence base while providing treatment effect estimates which can be included in decision models in a straightforward manner using techniques already widely in use.

In clinical areas such as oncology, epilepsy, and progressive diseases, RCTs often report both longitudinal and time-to-event outcomes. Methods have recently been developed that allow MA of joint longitudinal and time-to-event models [39]. Specific software packages for the fitting of joint models have been developed in both Stata [40] and R [41,42]. Specialist statistical expertise for the fitting of models and interpretation of results as well as specialist software are likely to be barriers to the uptake of joint models.

### 3.5 Nonproportional hazards

Conventionally, time-to-event outcomes are analyzed using a two-stage modeling approach in which the Cox model is used to obtain an estimate of the hazard ratio and its standard error for each trial in the first stage before synthesizing them using a fixed effect or random effects MA model in the second stage. The Cox model assumes proportional hazards over time, and it has been shown that this is not always a reasonable assumption, particularly in oncology when comparing chemotherapy and immunotherapy drugs [43]. In addition, clinicians can find hazard ratios hard to interpret [44]. Alternatives to the Cox model which can also allow for nonproportional hazards include fractional polynomial models [45], the piecewise exponential model [38,46], the Royston–Parmar model [47], and parametric survival curves [48]. Furthermore, a wider range of models can be fitted if a one-stage modeling approach is used. In the presence of nonproportional hazards, a single hazard ratio is no longer a suitable outcome measure. Alternative outcome measures, which have been used to synthesize evidence from multiple trials, include restricted mean survival time [49,50] and accelerated failure time [[51], [52], [53]]. The use of one-stage modeling approaches and alternative outcome measures is dependent on the data available from individual trials. Both one-stage models and restricted mean survival time require either individual participant data (IPD) or the reconstruction of IPD from Kaplan–Meier plots using methods such as the Guyot algorithm [54].

In our review of NIHR HTA reports, NICE technology appraisals, and NICE guidelines, the hazard ratio was the most commonly reported outcome measure for time-to-event outcomes. However, we also identified alternative outcome measures in almost a quarter of NICE technology appraisals, with the most popular method being fractional polynomial models. For example, in TA520, an NMA was conducted to assess the clinical effectiveness of atezolizumab compared with docetaxel and nintedanib to inform a cost-effectiveness analysis of atezolizumab. The proportional hazards assumption was tested and found to be violated, so a fractional polynomial approach was used for the NMA. Barriers to the uptake and implementation of methods for dealing with nonproportional hazards are likely to include specialist statistical expertise for the fitting of models and interpretation of results as well as the use of software such as R, Stata, or WinBUGS.

## 4 Implication of findings

From a combination of our review of NICE technology appraisals, NICE guidelines, and NIHR HTA reports and our expert knowledge of the relevant methodological area, we have identified five key areas where methodology is available for improving the synthesis of continuous and time-to-event outcomes within HTA. In the process, we have also identified what we believe to be are three barriers to the uptake and implementation of those methods:

### 4.1 Availability of specialist statistical expertise

Statistical expertise is advised for all MA and NMA; however, the previously described methods often require specialist statistical expertise in advanced evidence synthesis and/or survival analysis methodology to aid appropriate model selection, model fitting, and interpretation of results, which may not be available to all systematic review groups.

### 4.2 User-friendly software for implementing complex statistical and/or nonstandard models

The complex statistical and/or nonstandard models previously described often require either specific statistical software (e.g., WinBUGS) or development of bespoke statistical routines in packages such as R or Stata. For example, in TA520, the clinical effectiveness of atezolizumab was assessed using the fractional polynomial NMA model (as previously described). Cost-effectiveness was assessed using a three-state (on treatment, off treatment, and death) partitioned survival analysis model. The model was constructed by calculating the proportion of patients in each health state based on time-to-treatment discontinuation and overall survival curves, and the difference between the two curves, at discrete time points. Comparator curves for docetaxel and nintedanib were constructed by using atezolizumab as the reference and applying the time-dependent hazard ratios from the fractional polynomial NMA model. Fractional polynomials can be fitted in Stata, R, and WinBUGS but are not available in, the most commonly reported software used for synthesis, RevMan. Development of user-friendly software for implementing methods such as fractional polynomial NMA and other methods described in Section 3 may improve the uptake of these methods.

### 4.3 Limited reporting of individual trials

Limited reporting of outcomes data within individual trial publications/reports can restrict the choice of methods available for the analysis. For example, when using multivariate MA, a more precise estimate of the correlation between outcomes can be obtained if some trials report both outcomes. For all MA, a wider range of models are available if IPD is available for synthesis. Although we acknowledge that there is often a word limit for journal publications, we encourage researchers to publish analyses not included in the main article in online appendices and encourage the use of data repositories where possible.

## 5 Conclusion

Continuous and time-to-event outcomes are routinely synthesized within HTA articles. However, only approximately two-thirds of articles used the results of evidence synthesis to inform the decision model. Through our review of NIHR HTA reports, NICE technology appraisals, and NICE guidelines, we have identified five key areas where methodology is available for improving the synthesis of continuous and time-to-event outcomes: i) outcomes reported on multiple scales, ii) reporting of multiple related outcomes, iii) appropriateness of the additive scale, iv) reporting of multiple time points, and v) nonproportional hazards. In addition, we have identified three potential barriers to the uptake and implementation of these methods: i) availability of specialist statistical expertise for model selection, model fitting, and interpretation of results, ii) user-friendly software for implementing complex statistical and/or nonstandard models, and iii) limited reporting of individual trials. Addressing these barriers could increase the evidence base used within evidence syntheses of continuous and time-to-event outcomes and maximize the evidence base used in the decision making process. Therefore, it is important that analysts and decision modelers involved in the HTA process are aware of the expanding literature for the synthesis of continuous and time-to-event outcomes and appreciate the limitations of simpler approaches. Nevertheless, more research is needed to develop, refine, and generalize where possible methods for synthesizing continuous and time-to-event outcomes in this context for the purpose of decision modeling.

## CRediT authorship contribution statement

**Suzanne C. Freeman:** Conceptualization, Methodology, Software, Validation, Investigation, Data curation, Writing - original draft. **Alex J. Sutton:** Conceptualization, Methodology, Validation, Writing - review & editing. **Nicola J. Cooper:** Conceptualization, Methodology, Validation, Writing - review & editing.

## Appendix A Supplementary data

Data Profile
