# Supplementary material for: Uptake of methodological advances for synthesis of continuous and time-to-event outcomes would maximize use of the evidence base
Source: J Clin Epidemiol. 2020 Aug;124:94–105. doi: 10.1016/j.jclinepi.2020.05.010 (PMC7435685; doi:10.1016/j.jclinepi.2020.05.010)
Supplement: Appendix [file mmc2.docx]

**Appendix A: Data Extraction**

Pre-specified items:

- Title
- Type of article (NICE Technology Appraisal, NICE Guideline, NIHR HTA Report)
- Month and year published
- Primary outcome
- Secondary outcomes
- Continuous outcome – yes/no
- Time-to-event outcome – yes/no
- Outcome measure
- Is a meta-analysis conducted?
- Is a network meta-analysis conducted?
- Did they only include randomised controlled trials in the evidence synthesis?
- Fixed or random effects
- Was there any analysis of multiple outcomes simultaneously?
- Was there lumping of interventions?
- Were multiple pairwise comparisons reported for the primary outcome?
- Was there any standardisation of outcomes? If so, how?
- Were multiple time points analysed? If so, how?
- Was there a network diagram?
- Were the results of synthesis presented as forest plots?
- Were the results of synthesis presented as Kaplan-Meier plots?
- Were the results of synthesis reported in tables?
- Were any other methods used to present the results of synthesis? If so, what were they?
- Was an economic decision model presented?
- Did clinical results inform the economic decision model?
- Were the results of the evidence synthesis used in the economic decision model?
- How much clinical evidence informed the economic decision model?
- What was the source of the clinical evidence for the economic decision model?
- What analysis method was used for the economic decision model?
- Was there a graphical display of the economic decision model?
- Software used for clinical effectiveness
- Software used for cost effectiveness

Items collected following peer review:

- Clinical field
- Type of intervention (pharmacological, non-pharmacological, both)

**Appendix B: List of reviewed articles**

**NIHR HTA Reports**

Lamb SE, Mistry D, Alleyne S, Atherton N, Brown D, Copsey B, Dosanjh S, Finnegan S, Fordham B, Griffiths F, Hennings S, Khan I, Khan K, Lall R, Lyle S, Nichols V, Petrou S, Zeh P, Sheehan B, on behalf of the DAPA trial group. Aerobic and strength training exercise programme for cognitive impairment in people with mild to moderate dementia: The DAPA RCT. Volume 22, Issue 28, May 2018.

Thompson SG, Bown MJ, Glover MJ, Jones E, Masconi KL, Michaels JA, Powell JT, Ulug P, Sweeting MJ. Screening women aged 65 years or over for abdominal aortic aneurysm: A modelling study and health economic evaluation. Volume 22, Issue 43, August 2018.

Mujica-Mota R, Varley-Campbell J, Tikhonova I, Cooper C, Griffin E, Hassova M, Peters J, Lucherini S, Talens-Bou J, Long L, Sherriff D, Napier M, Ramage J, Hoyle M. Everolimus, lutetium-177 DOTATATE and sunitinib for advanced, unresectable or metastatic neuroendocrine tumours with disease progression: A systematic review and cost-effectiveness analysis. Volume 22, Issue 49, September 2018.

Beresford B, McDaid C, Parker A, Scantlebury A, Spiers G, Fairhurst C, Hewitt C, Wright K, Dawson V, Elphick H, Thomas M. Pharmacological and non-pharmacological interventions for non-respiratory sleep disturbance in children with neurodisabilities: A systematic review. Volume 22, Issue 60, October 2018.

Avenell A, Robertson C, Skea Z, Jacobsen E, Boyers D, Cooper D, Aceves-Martins M, Retat L, Fraser C, Aveyard P, Stewart F, MacLennan G, Webber L, Corbould E, Xu B, Jaccard A, Boyle B, Duncan E, Shimonovich M, de Bruin M. Bariatric surgery, lifestyle interventions and orlistat for severe obesity: The REBALANCE mixed-methods systematic review and economic evaluation. Volume 22, Issue 68, November 2018.

Snowsill T, Yang H, Griffin E, Long L, Varley-Campbell J, Coelho H, Robinson S, Hyde C. Low-dose computed tomography for lung cancer screening in high-risk populations: A systematic review and economic evaluation. Volume 22, Issue 69, November 2018.

Tappenden P, Carroll C, Hamilton J, Kaltenthaler E, Wong R, Wadsley J, Moss L, Balasubramanian S. Cabozantinib and vandetanib for unresectable locally advanced or metastatic medullary thyroid cancer: A systematic review and economic model. Volume 23, Issue 8, February 2019.

**NICE Technology Appraisals**

TA520: Atezolizumab for treating non-small-cell lung cancer after platinum-based chemotherapy. May 2018.

TA521: Guselkumab for treating moderate to severe plaque psoriasis. June 2018.

TA522: Pembrolizumab for untreated PD-L1-positive locally advanced or metastatic urothelial cancer when cisplatin is unsuitable. June 2018.

TA525: Atezolizumab for treating locally advanced or metastatic urothelial carcinoma after platinum-containing chemotherapy. June 2018.

TA530: Nivolumab for treating locally advanced unresectable or metastatic urothelial cancer after platinum-containing chemotherapy. July 2018.

TA533: Ocrelizumab for treating relapsing–remitting multiple sclerosis. July 2018.

TA534: Dupilumab for treating moderate to severe atopic dermatitis. August 2018.

TA535: Lenvatinib and sorafenib for treating differentiated thyroid cancer after radioactive iodine. August 2018.

TA537: Ixekizumab for treating active psoriatic arthritis after inadequate response to DMARDs. August 2018.

TA538: Dinutuximab beta for treating neuroblastoma. August 2018.

TA540: Pembrolizumab for treating relapsed or refractory classical Hodgkin lymphoma. September 2018.

TA542: Cabozantinib for untreated advanced renal cell carcinoma. October 2018.

TA543: Tofacitinib for treating active psoriatic arthritis after inadequate response to DMARDs. October 2018.

TA545: Gemtuzumab ozogamicin for untreated acute myeloid leukaemia. November 2018.

TA554: Tisagenlecleucel for treating relapsed or refractory B-cell acute lymphoblastic leukaemia in people aged up to 25 years. December 2018.

TA557: Pembrolizumab with pemetrexed and platinum chemotherapy for untreated, metastatic, non-squamous non-small-cell lung cancer. January 2019.

TA558: Nivolumab for adjuvant treatment of completely resected melanoma with lymph node involvement or metastatic disease. January 2019.

TA559: Axicabtagene ciloleucel for treating diffuse large B-cell lymphoma and primary mediastinal large B-cell lymphoma after 2 or more systemic therapies. January 2019.

TA561: Venetoclax with rituximab for previously treated chronic lymphocytic leukaemia. February 2019.

TA562: Encorafenib with binimetinib for unresectable or metastatic BRAF V600 mutation-positive melanoma. February 2019.

TA563: Abemaciclib with an aromatase inhibitor for previously untreated, hormone receptor-positive, HER2-negative, locally advanced or metastatic breast cancer. February 2019.

TA565: Benralizumab for treating severe eosinophilic asthma. March 2019.

TA567: Tisagenlecleucel for treating relapsed or refractory diffuse large B-cell lymphoma after 2 or more systemic therapies. March 2019.

TA571: Brigatinib for treating ALK-positive advanced non-small-cell lung cancer after crizotinib. March 2019.

TA572: Ertugliflozin as monotherapy or with metformin for treating type 2 diabetes. March 2019.

**NICE Guidelines**

NG95: Lyme disease. April 2018.

NG97: Dementia: assessment, management and support for people living with dementia and their carers. June 2018.

NG98: Hearing loss in adults: assessment and management. June 2018.

NG99: Brain tumours (primary) and brain metastases in adults. July 2018.

NG100: Rheumatoid arthritis in adults: management. July 2018.

NG101: Early and locally advanced breast cancer: diagnosis and management. July 2018.

NG102: Community pharmacies: promoting health and wellbeing. August 2018.

NG104: Pancreatitis. September 2018.

NG105: Preventing suicide in community and custodial settings. September 2018.

NG106: Chronic heart failure in adults: diagnosis and management. September 2018.

NG107: Renal replacement therapy and conservative management. October 2018.

NG115: Chronic obstructive pulmonary disease in over 16s: diagnosis and management. December 2018.

NG116: Post-traumatic stress disorder. December 2018.

NG118: Renal and ureteric stones: assessment and management. January 2019.

NG119: Cerebral palsy in adults. January 2019.

**Appendix C: Reported Outcomes**

Table C.1 Outcomes reported in articles synthesising continuous outcomes

|  | **NICE Technology Appraisal** | **NICE Guidelines** | **NIHR HTA Report** |
| --- | --- | --- | --- |
| **Outcome** | **7** | **13** | **4** |
| Objective response rate | 1 | 0 | 0 |
| Psoriatic arthritis response criteria (PsARC) | 2 | 0 | 0 |
| Scoring atopic dermatitis (SCORAD) | 1 | 0 | 0 |
| Psoriasis area and severity index | 1 | 0 | 0 |
| Percentage reduction from baseline steroid use | 1 | 0 | 0 |
| Change in HbA1c | 1 | 0 | 0 |
| Health related quality of life | 0 | 4 | 0 |
| Change in trough FEV1 | 0 | 1 | 0 |
| Time to stone passage | 0 | 1 | 0 |
| Change in PTSD symptoms | 0 | 1 | 0 |
| Suicide rate | 0 | 1 | 0 |
| Mini mental state examination | 0 | 1 | 0 |
| PTA (puretone average) final score | 0 | 1 | 0 |
| Weight change | 0 | 1 | 1 |
| Change in disease activity score | 0 | 1 | 0 |
| 11 item fatigue severity scale score at 24 weeks | 0 | 1 | 0 |
| Quality of sleep | 0 | 0 | 1 |
| Percentage of patients EVAR (endovascular treatment of abdominal aortic aneurysm) suitable | 0 | 0 | 1 |
| Alzheimer’s disease assessment scale cognitive subscale | 0 | 0 | 1 |

Table C.2 Outcomes reported in articles synthesising time-to-event outcomes

|  | **NICE Technology Appraisal** | **NICE Guidelines** | **NIHR HTA Report** |
| --- | --- | --- | --- |
| **Outcome** | **19** | **4** | **3** |
| Overall survival | 9 | 3 | 2 |
| Progression free survival | 6 | 0 | 1 |
| Event free survival | 2 | 0 | 0 |
| Recurrence free survival | 1 | 0 | 0 |
| Disease free survival | 0 | 1 | 0 |
| Annualised relapse rate | 1 | 0 | 0 |
